# Antiquities in New Mexico

**Published:** 1881-06

**Authors:** 


### ANTIQUITIES IN NEW MEXICO.

The walls of some of the old ruins at Abo are six feet of solid stone—lime and red sand ; the walls in places are yet six feet in height, and in a state of perfect preservation. In the ruins are found vessels of various designs and sizes made of pottery, some representing birds and animals. Stone hammers are found there, but no indication that sharp-edged tools were used in this ancient period. In digging down one place the remains of an old aqueduct were found, which was probably used, as in the present day, by the Mexicans for supplying the inhabitants with water.

It is thought and believed, from specimens of ore found, that gold, silver, and copper were found in paying quantities. All the rock is more or less copper stained, and some of it is so much so that some of the "country" rock has run as high as 37 per cent. copper.

Surely our bright, sunny land has been enjoyed long before the Anglo-Saxon made his appearance upon the scene. The future of New Mexico can only be surmised.

New Mexico is perhaps the most noted country in the world for research. The historian, the wealth-seeker and the "curious" can here find a rich field and reward for their labor. The Abo and Gran Quivira counties are perhaps the most renowned in the territory for research. In the former there are evidences of great volcanic eruptions which overwhelmed cities and buried the inhabitants in ashes and lava long ages ago. It is evident that these people, who are perhaps older than the Aztecs, were a prosperous race, with not a little advance in civilization, as the Abo ruins in the Manzana

Mountains indicate ; also some indications of fine art ; rude figures and the images of animals being found upon the interior of the walls of the structures beneath the *debris.*

It is evident that this non-historic race were seekers after mineral, and evidences also exist that mineral was obtained by them in paying quantities, there being the ruins of many old smelters and acres of slag found near Abo. Here mines are found with the timbers so rotten with age that great difficulty is experienced and danger incurred in going down into the old shafts, where shafts are formed.—*Kansas City Review.*

